# Enrichment and Detection of Antigen-Binding B Cells for Mass Cytometry

**DOI:** 10.3390/magnetochemistry7070092

**Published:** 2021-06-23

**Authors:** Zachary C. Stensland, Mia J. Smith

**Affiliations:** 1 Barbara Davis Center for Diabetes, Department of Pediatrics, University of Colorado School of Medicine, Aurora, CO 80045, USA;; 2 Department of Immunology and Microbiology, University of Colorado School of Medicine, Aurora, CO 80045, USA

**Keywords:** CyTOF, mass cytometry, magnetic enrichment, antigen-specific B cells, antigen-binding B cells, antigen-reactive B cells, B lymphocytes, B cells

## Abstract

Over the years, various techniques have been utilized to study the function and phenotype of antigen-binding B cells in the primary repertoire following immunization, infection, and development of autoimmunity. Due to the low frequency of antigen-reactive B cells (<0.05% of lymphocytes) in the periphery, preliminary enrichment of cells is necessary to achieve sufficient numbers for statistically sound characterization, especially when downstream analytic platform use, e.g., CyTOF, is low throughput. We previously described a method to detect and enrich antigen-reactive B cells from peripheral blood and tissues using biotinylated antigens in conjunction with magnetic nanoparticles, preparative to a downstream analysis by ELISPOT and flow cytometry. While mass cytometry (CyTOF) enables high dimensional immunophenotyping of over 40 unique parameters on a single-cell level, its low throughput compared to flow cytometry and requirement for removal of metal contaminants, such as nanoparticles, made it particularly unsuitable for studies of rare cells in a mixed population. Here we describe a novel CyTOF-compatible approach for multiplexed enrichment of antigen-reactive B cells, e.g., insulin and tetanus toxoid, using cleavable magnetic nanoparticles. This method allows improved monitoring of the phenotype and function of antigen-reactive B cells during the development of disease or after immunization while minimizing the amount of sample and run times needed.

## 1. Introduction

Effective study of the biology of antigen-reactive B cells before and during the development of the immune responses and their perturbation during disease development requires examination of B cells on an antigen-specific level. Previous methods to identify antigen-reactive B cells have included assays such as limiting dilution, ELISPOT, and most commonly flow cytometry [1–9]. Previously, we developed a method to identify and enrich rare antigen-reactive B cells from polyclonal repertoires using biotinylated antigen and magnetic nanoparticles, which is compatible with a variety of downstream assays [10]. Using this method, we studied changes in the phenotype of self-reactive B cells during the development of type 1 diabetes and autoimmune thyroid disease, as well as foreign-reactive (e.g., tetanus) B cells prior to and after vaccination [10–14]. While our previous method has been instrumental in better understanding phenotypic changes in antigen-reactive B cells during the course of the disease and after vaccination, it is limited by the relatively low number of parameters able to be assayed simultaneously by flow cytometry (even with spectral flow cytometry).

Mass cytometry, or CyTOF (Fluidigm), is a technology that combines flow cytometry and mass spectrometry in which antibodies or other ligands used to label cells are conjugated to heavy metal isotopes that are identified using time-of-flight mass spectrometry [15,16]. Some advantages of mass cytometry over traditional fluorescence-based flow cytometry are that currently as many as 50 individual parameters can be analyzed simultaneously with minimal spillover between output signals and little need for compensation or unmixing. Like flow cytometry, mass cytometry can be used to study the phenotypic and functional properties of various cell types thanks to its compatibility with pre-existing methods such as immunophenotyping, intracellular staining, and phosphoflow. Moreover, it was recently demonstrated that mass cytometry is comparable to flow cytometry in terms of quantification of cell lineages and markers of activation, function, and exhaustion using low numbers of human PBMCs [17].

Hence, in order to take advantage of the high-parameter single-cell immunophenotyping capability of mass cytometry, we developed a method to enable enrichment and identification of antigen-binding B cells from human PBMCs that is compatible with mass cytometry. During the initial development of this method, we first attempted to identify antigen-reactive B cells using directly metal labeled antigens that bind to the B cell receptor (BCR) followed by CyTOF analysis, without intervening enrichment. However, we found that due to the low throughput of mass cytometry statistically sound characterization of antigen-reactive B cells using mass cytometry necessitates prior enrichment of the cells of interest. However, unlike flow cytometry, for this analysis, magnetic nanoparticles must be removed from enriched cells because they contain barium and other heavy metals that may foul the spectrometer. This can be accomplished using “cleavable” beads, as previously described [18]. Hence, in our method, we utilize commercially available cleavable anti-biotin and anti-FITC magnetic beads to enrich our antigen-reactive B cells of interest. In addition, after careful optimization, we have modified our previous method to enable the identification and tracking of more than one type of antigen-reactive B cell within the same sample. Importantly, while successful identification of antigen-specific T cells has been previously shown using mass cytometry [18–20], to our knowledge, this is the first study to demonstrate the ability to characterize antigen-reactive B cells using this tool. Moreover, previous studies to analyze antigen-specific T cells using mass cytometry have either, (1) used metal labeled tetramers to detect antigen-specific T cells without prior enrichment, resulting in very few cells of interest for analysis, or (2) enriched for antigen-reactive T cells based on upregulation of activation markers (e.g., CD69) after stimulation with antigen in vitro. Hence, our method is unique in that it combines detection using the antigen of interest as bait with enrichment using cleavable beads, resulting in a more pure population for analysis.

**Figure 1.**
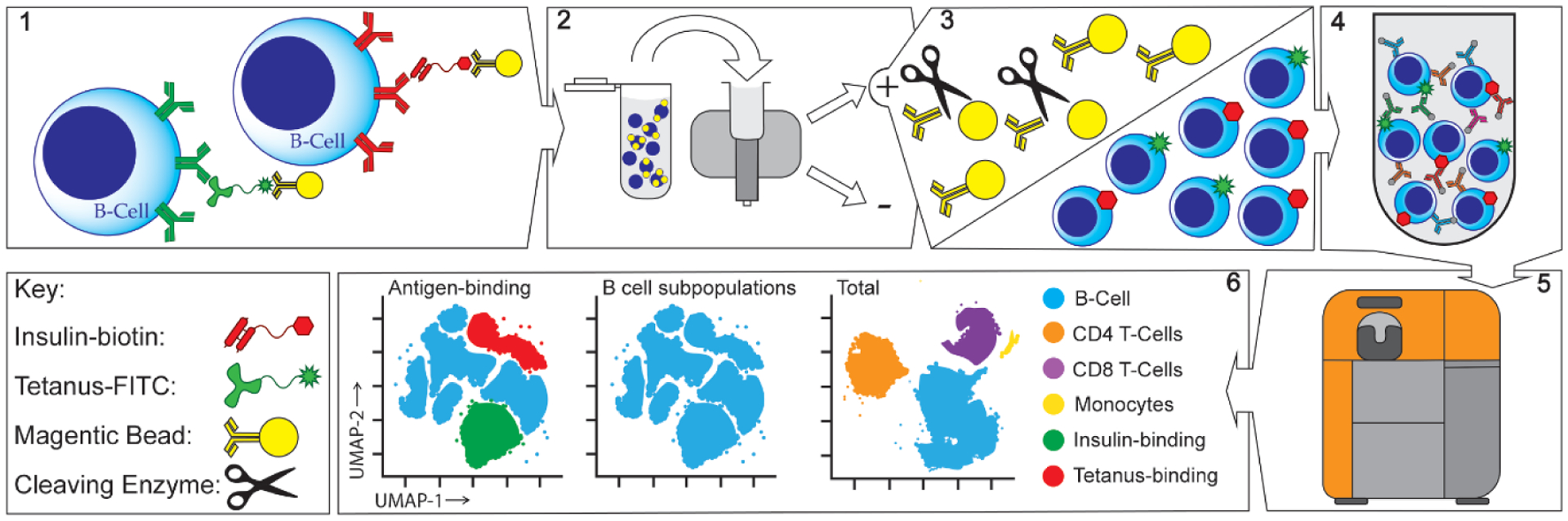
Schematic for magnetic enrichment of antigen-reactive B cells. 1. Human PBMCs are incubated with properly titrated amounts of biotin and FITC-labeled antigens. Cells are then incubated with a combination of cleavable anti-biotin and anti-FITC magnetic beads. 2. Washed cells are then added to a magnetic column and separated into depleted and enriched fractions. 3. Magnetic microbeads are cleaved off and washed away. 4. Cells are then incubated with an antibody cocktail containing various metal labeled antibodies. 5. Cells are analyzed on a mass cytometer. 6. Data analysis is performed using a combination of manual gating and dimensionality reduction and clustering algorithms.

Here we demonstrate simultaneous enrichment of insulin-binding B cells and tetanus-toxoid-binding B cells from the peripheral blood of healthy individuals using mass cytometry. The basic steps for our protocol are as follows: first, PBMCs are incubated with a live-dead marker [21], followed by staining with FITC conjugated tetanus-toxoid and biotinylated insulin. Cells are fixed, followed by enrichment of antigen-binding B cells using cleavable anti-biotin and anti-FITC magnetic beads and LS MACS columns (Miltenyi Biotech, Bergisch Gladbach, Germany). The magnetic beads are then cleaved away from labeled cells. After washing, the enriched cells are stained with metal-labeled antibodies to various cell surface markers, as well as metal labeled anti-FITC and anti-biotin antibodies to identify tetanus and insulin-binding B cells, respectively. Finally, enriched cells are analyzed by the mass cytometer. A schematic for this method is represented in Figure 1 and the materials needed are listed in Table 1. We conclude with a discussion of the various limitations of the method and important considerations that should be taken into account when applying the method.

## 2. Materials and Methods

### Protocol:

1. Isolation of PBMC’s from Whole Blood
  1. Collect 30–50 mL of heparinized blood collection tubes
  2. Dilute heparinized blood 1:1 with sterile, room temperature magnesium and calcium-free phosphate-buffered saline (PBS).
  3. In a 50 mL conical tube, add 10–15 mL of room temperature Ficoll-Paque PLUS.
  4. Carefully layer 30–35 mL of the diluted blood on the top of the Ficoll-Paque at the lowest speed setting, being careful not to mix the two.
  5. Centrifuge at 400× *g* for 30 min at room temperature with the brake turned off.
  6. Using a serological pipette remove the mononuclear layer (buffy coat) and transfer to a clean 50 mL conical. Add at least 3 times the volume of PBS to the cell suspension.
  7. Centrifuge cells at 400× *g* for 10 min at room temperature with the brake on.
  8. Remove supernatant and resuspend cells in 10 mL PBS. Count cells and determine viability using a hemocytometer or automated cell counter.
  9. Adjust cell concentration to 3 × 10^7^ cells/1 mL CSM buffer and place on ice until ready to begin processing.
2. Viability Dye
  1. Centrifuge 30 × 10^6^ cells at 400× *g* for 5 min at room temperature and aspirate supernatant.
  2. Wash cells 1× with 1 mL sterile PBS
    - Do not use any solutions with serum or protein content until after cisplatin incubation.
  3. Spin down at 400× *g* for 5 min at room temperature and aspirate supernatant.
  4. Resuspend cells in serum-free RPMI at 3 × 10^6^ cells per 1 mL RPMI.
  5. Add 1 uL (25 mM stock) of Cisplatin per 1 mL of serum-free RPMI and vortex samples briefly to mix thoroughly (25 uM final concentration).
  6. Incubate for 1 min at RT before quenching solution with 2 mL CSM or complete RPMI per 1 mL of cisplatin-cell solution.
    - It is necessary to quench with a solution that contains protein to bind extraneous cisplatin
  7. Spin cells at 400× *g* for 5 min at room temperature and aspirate supernatant.
3. Enrichment of antigen-reactive B cells
  1. Wash cells once with 1 mL CSM. Centrifuge cells at 400× *g* for 5 min and aspirate supernatant.
  2. Resuspend cells in 25 uL Fc Block and allow them to bind for 5 min on ice. Do not wash off.
  3. Add 1 mL CSM to cells before adding antigen(s) at their pre-determined amount. Vortex briefly to mix and incubate on ice for 15 min in the dark.
    - Note: Biotinylated and FITC conjugated antigens should be titrated to determine the optimal concentrations needed to ensure detection and separation of binding cells from non-specific bystander cells. Typically, a good starting concentration is between 0.025 ug and 1 ug per 1 × 10^6^ cells. (See Smith et al. for detailed protocol [10])
    - Eagle-Insulin-biotin (0.4 mg/mL stock, commercially available): Use 0.035 ug/1 × 10^6^ cells = 1.05 ug/ 30 × 10^6^ cells = 2.63 uL
    - Tetanus-Toxoid-FITC (2 mg/mL stock, FITC labeled according to manufacturer’s instructions): Use 1 ug/1 × 10^6^ cells = 30 ug /30 × 10^6^ cells. = 15 uL
  4. Wash cells 2× with 1 mL cold CSM at 400× *g* for 5 min before aspirating supernatant.
  5. During centrifugation prepare 1.6% PFA solution using the following steps:
    - Break open an unopened ampule of 16% paraformaldehyde and sterile filter using a 0.45 μm syringe filter into a 15 mL conical tube.
    - Dilute to 1.6% PFA by adding 9 mL of CSM to the 1 mL of 16% PFA. Wrap conical tightly in tin foil to avoid light exposure. Keep ~1 week.
    - It is important to use fresh PFA, as improperly fixed cells can lyse after the addition of MilliQ water.
  6. Aspirate supernatant and resuspend the cells in 1 mL 1.6% PFA. Allow for staining in dark at room temperature for 5 min.
  7. Wash cells 2× with 1 mL cold CSM at 400× *g* for 5 min before aspirating supernatant.
  8. Resuspend cells in the following:
    - 60 uL MACS cleavable anti-biotin beads (20 uL/10^7^ cells) + 30 uL MACS cleavable anti-FITC beads (10 uL/10^7^ cells) + 210 uL CSM (final volume should be 100 uL/10^7^ cells)
      1. The amount of anti-biotin and anti-FITC beads to add should be determined beforehand using the manufacturer’s instructions.
  9. Vortex briefly to mix and incubate cells for 15 min at 4 °C in the dark.
  10. Add 1 mL cold CSM to the mixture and centrifuge at 400× *g* for 5 min. Aspirate the supernatant.
  11. During this time, place MACS LS column on MACS magnet. Allow 3 mL of cold CSM to flow through the column to prime the column.
  12. Resuspend cells in 1 mL CSM and add to LS column. Collect “depleted” flow through in 15 mL conical tube.
  13. Wash unbound cells from the column by adding 3 mL cold CSM on top of the LS column and allowing the volume to completely flow through the column into the depleted fraction. Repeat with another 3 mL of cold CSM.
  14. Place a new 15 mL conical tube labeled “enriched” under the LS column and fill with 6 mL of cold CSM. Plunge the CSM through the column using equal steady pressure to collect cells held in the magnetic field using the provided plunger.
  15. Centrifuge both depleted and enriched fractions of cells at 400× *g* for 10 min at 4 °C.
  16. Resuspend the depleted fraction in an appropriate volume to re-count. Resuspend 2–3 × 10^6^ cells from the depleted fraction in 1 mL cold CSM in a 1.5 mL Eppendorf tube. Resuspend the enriched fraction in 1 mL cold CSM in a 1.5 mL Eppendorf tube (we assume the number of cells in our enriched fraction is ≤1 million cells).
  17. Add 20 uL multi-sort release agent per 1 mL cell suspension. Vortex briefly to mix and incubate for 10 min at 4 °C in the dark.
  18. Wash cells by adding 9–10 mL cold CSM to the cell suspension and centrifuge at 400× *g* for 10 min at 4 °C.
  19. Aspirate supernatant and wash again in 1 mL cold CSM at 400× *g* for 10 min at 4 °C.
  20. Partially aspirate supernatant leaving approximately 50 uL of CSM in the tube.
4. Surface Staining
  1. Create 100 uL 2X antibody master mix (MM) of surface antibodies from Table 2 in CSM.
    - Note: The optimal amount of each metal labeled antibody to add should be determined beforehand.
    - Add 2 uL each of 160Gd-anti-FITC and 170Er-anti-biotin antibodies to the MM to detect tetanus and insulin-reactive B cells, respectively.
  2. Add 50 uL of MM to the depleted sample and 50 uL MM to the enriched sample, such that the final concentration of antibodies per sample is 1X. Vortex briefly and incubate on ice for 15–20 min in dark.
  3. Add 1 mL CSM to samples and centrifuge at 400× *g* for 5 min.
  4. Aspirate supernatant and resuspend in 100 uL of 1.6% PFA. Allow cells to stain in dark for 5 min. Add 1 mL CSM and centrifuge at 400× *g* for 5 min.
  5. While spinning, create an intercalator cocktail:
    - 1 mL 1.6% PFA + 0.5 uL intercalator IR per sample.
  6. Add 1 mL of intercalator solution to each sample and leave at 4 °C for a minimum of 20 min or up to 1 week.
  7. Wash 3× in MilliQ water.
  8. Resuspend in 1 mL MilliQ water per million cells for running on mass cytometer.
  9. Data acquisition and analysis
  10. Acquire data on mass cytometer, as previously described [21].
  11. Analyze data using third-party analysis software, such as FlowJo (Treestar) or CellEngine [22].

## 3. Representative Results

**Figure 2.**
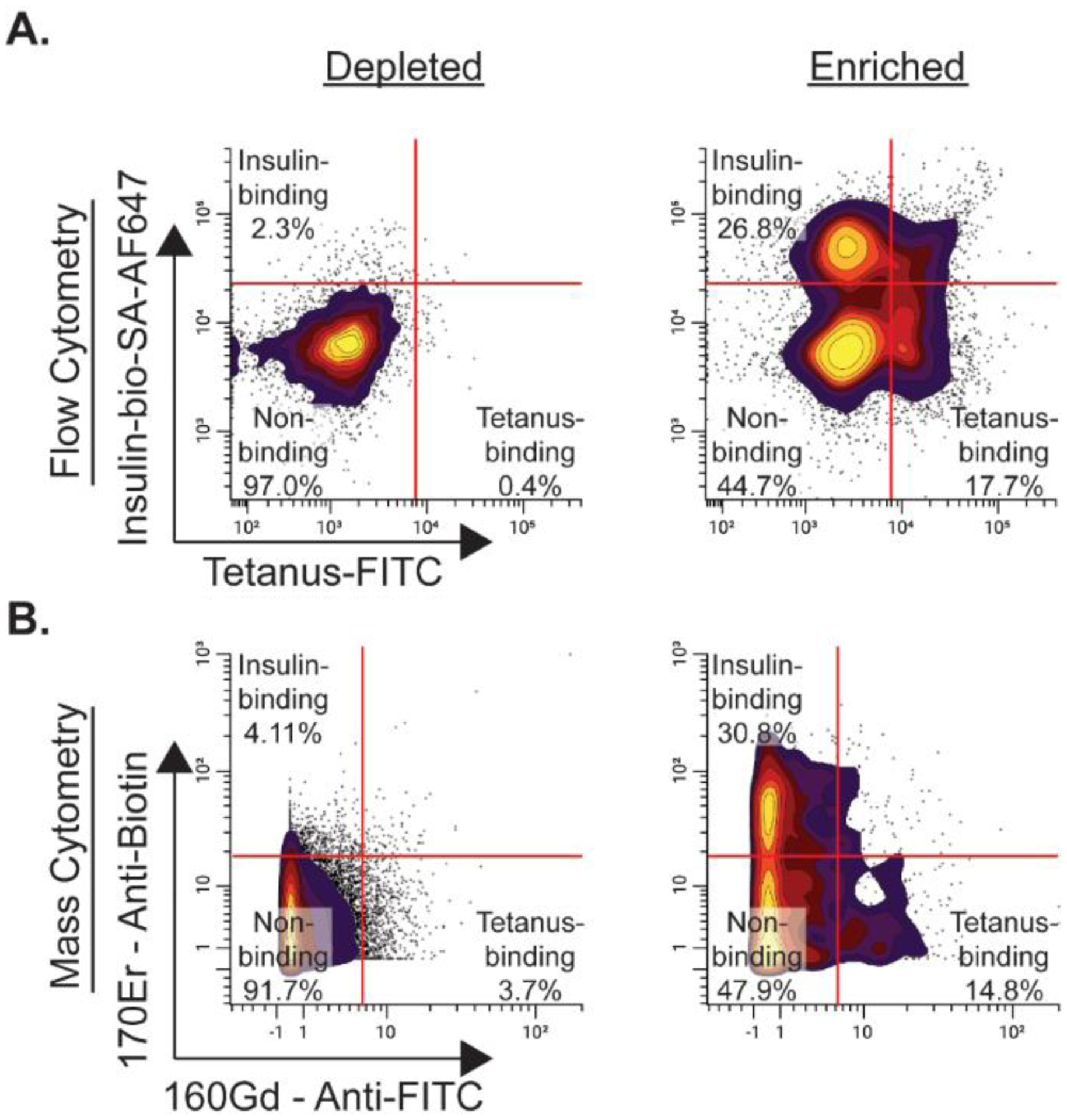
Representative comparison of enrichment of insulin and tetanus-binding B cells using flow cytometry and mass cytometry shows similar binding cell frequencies. Approximately 60 million PBMCs were collected from a single human donor, split in half, with each half enriched for insulin and tetanus-binding B cells as described in this protocol. (**A**) Representative binding of insulin and tetanus-binding B cells of depleted and enriched fractions using flow cytometry. (**B**) Representative binding of insulin and tetanus-binding B cells using mass cytometry. For the flow cytometry sample, the addition of fluorescently labeled antibodies (BV421 anti-CD19 and PerCP anti-CD3) and Streptavidin-AlexaFluor647 to detect biotinylated insulin bound to the BCR were incubated with the cells in place of metal-labeled antibodies and secondaries. Depleted fractions are negatively enriched cells and enriched fractions are positively enriched cells. Data were analyzed using CellEngine.

To demonstrate the method described above, we show representative mass cytometry data obtained using FITC labeled tetanus-toxoid and biotinylated insulin as model antigens to detect and enrich simultaneously for foreign and self-reactive B cells, respectively, from the peripheral blood of a healthy individual. Figure 1 is an overall schematic of the method. Figure 2 shows a comparison of the method using flow cytometry and mass cytometry starting from ~60 million fresh PBMCs from the same donor that were divided in half and enriched as described above with fluorescently labeled markers substituted for the flow cytometry sample. The donor was last vaccinated with tetanus-toxoid more than 2 years prior to the analysis. Insulin and tetanus-binding B cells were found in frequencies similar to those observed by flow cytometry. Hence, antigen-binding cells are not differentially lost with mass cytometry. However, cell recovery was reduced with mass cytometry compared to flow cytometry, as expected. For the flow cytometry sample, there were approximately 3000 insulin-binding B cells and 2000 tetanus-binding B cells identified, and for the mass cytometry sample, there were approximately 1000 insulin-binding B cells and 500 tetanus-binding B cells identified. Despite the cell loss, antigen-binding B cells can still be easily analyzed for expression of other markers to distinguish varying B cell subpopulations and expression of cell surface markers.

**Figure 3:**
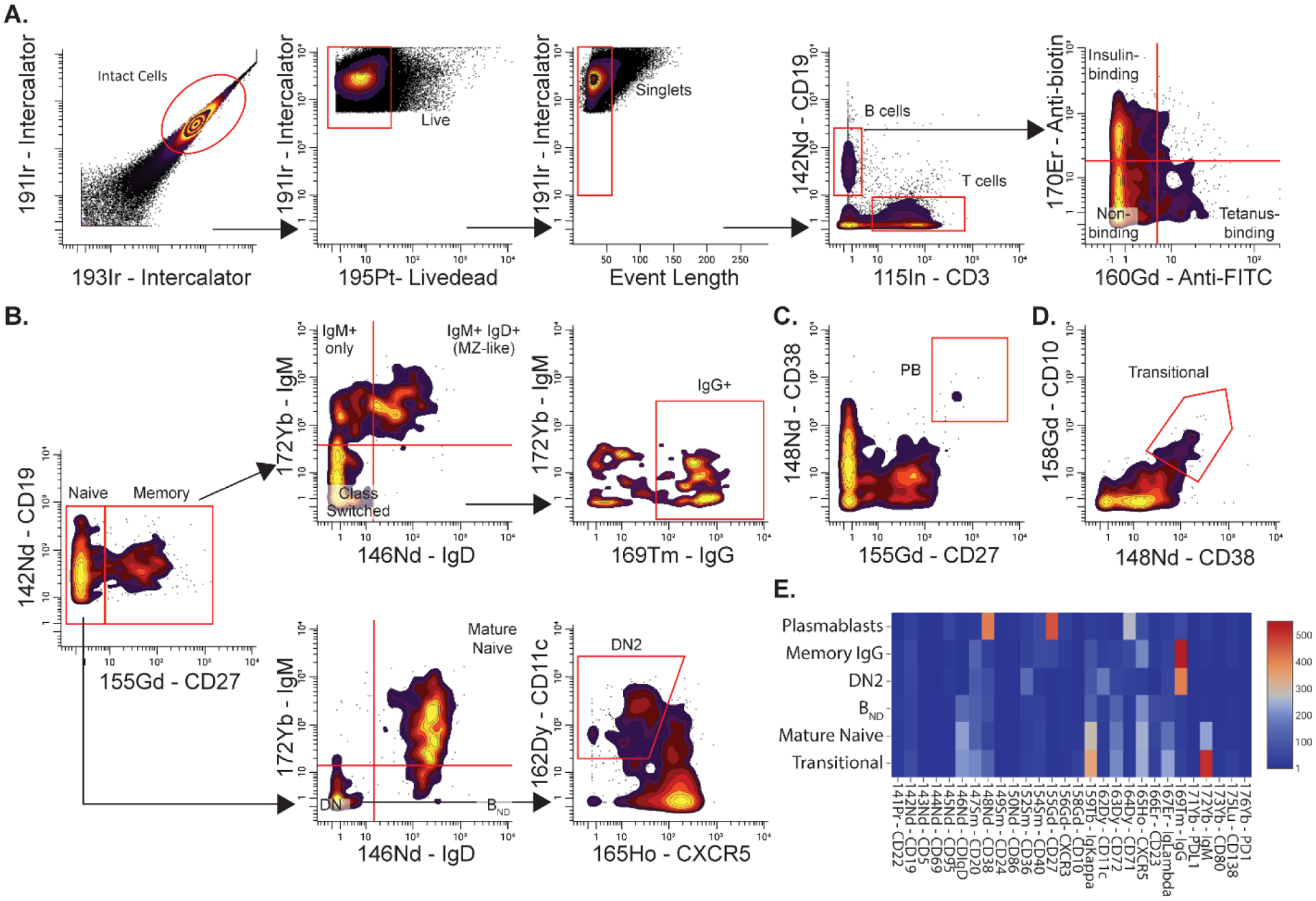
Representative manual gating strategy for identification of antigen-binding B cells and major B cell subsets. (**A**) Initial gating on intact cells, live cells, singlets, and then B cells to identify insulin and tetanus-binding B cells using mass cytometry. (**B**) Representative gating to identify major B cell subjects, including naïve (CD27−), memory (CD27+), CS-memory (CD27+ IgM− IgD−), Marginal zone (MZ) memory (CD27+ IgM+ IgD+), IgG+ memory (CD27+ IgG+), mature naïve (CD27− IgM+ IgD+), anergic B_ND_ (CD27− IgMlo IgD+), double-negative (DN) (CD27− IgD−), and DN2 (CD27− IgD− CXCR5− CD11c+). Cells were initially gated on CD19+ cells. (**C**) Representative gating to identify plasmablasts (CD27hi CD38hi). Cells were initially gated on CD19+ cells. (**D**) Representative gating to identify transitional B cells (CD10hi CD38hi). Cells were initially gating on naïve B cells (CD19+ CD27−). (**E**) Representative heatmap of various cell surface markers expressed on manually gated B cell subsets. Colors represent raw mean metal intensities (MMI). Data were analyzed by CellEngine.

Figure 3 demonstrates a representative manual gating strategy used to define major B cell subsets and analysis of cell surface expression of various markers using mass cytometry (Table 2). Figure 3A shows representative gating used to identify insulin, tetanus, and non-binding B cells. We have previously shown that the use of magnetic beads to enrich antigen-binding B cells inevitably results in a number of non-binding bystander cells that get trapped in the matrix [10]. While these impurities can be removed by FACS sorting, non-binding cells represent a good internal negative control to determine differences between non-binding and binding B cells. Figure 3B–D demonstrate representative gating to identify major B cell subjects, including naïve, memory, IgG+ memory, double-negative (DN), anergic (B_ND_), DN2, plasmablast, and transitional B cells. Once major B cell subpopulations are identified using manual gating strategies, heat maps expressing the mean metal intensities (MMI) of various cell surface molecules can be generated to identify populations that may be highly activated or have altered expressions of varying surface markers (Figure 3E).

**Figure 4.**
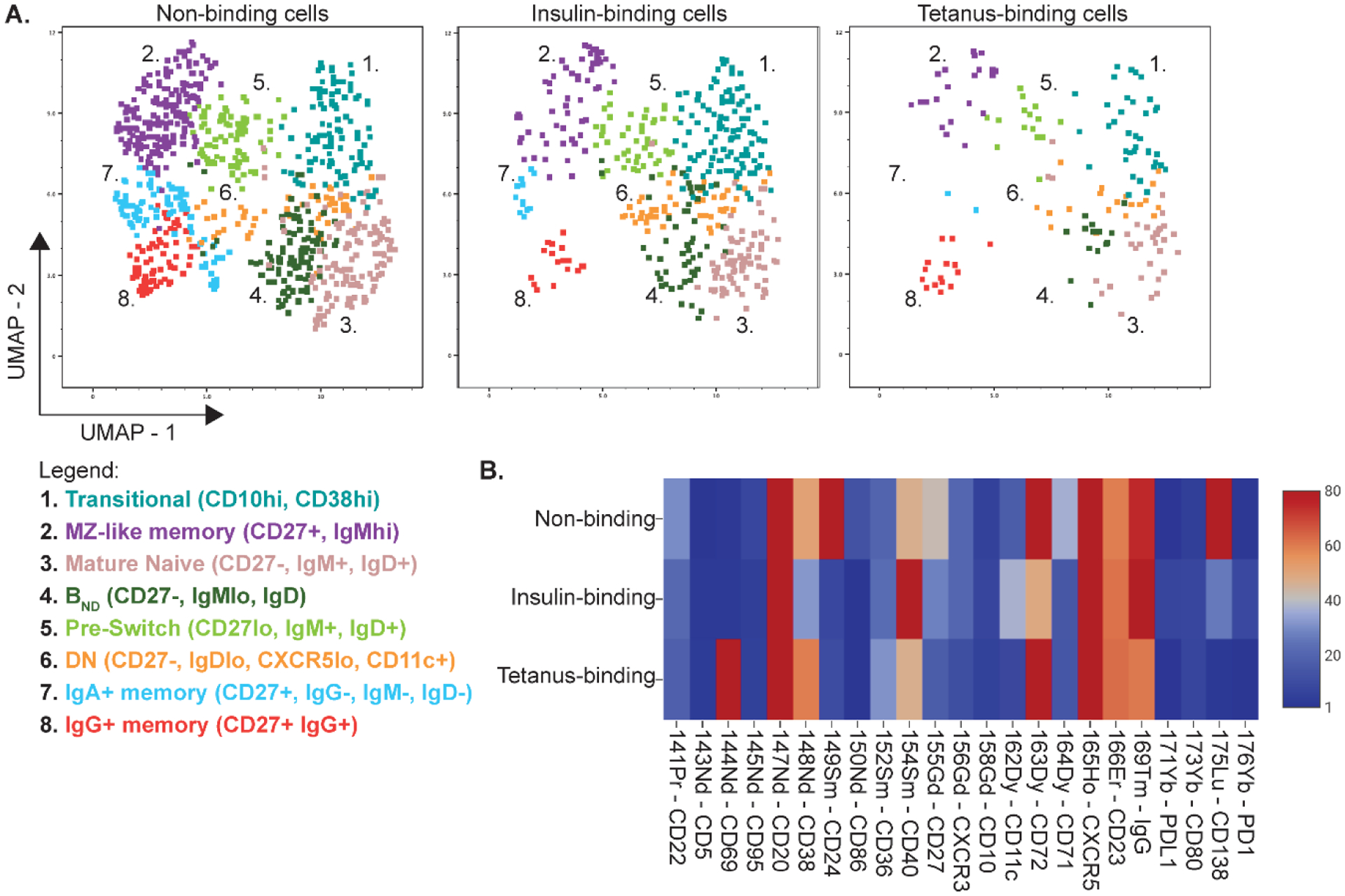
Representative UMAP projections of various B cell subpopulations within the insulin, tetanus, and non-binding B cells demonstrate unique B cell compositions within each antigen-binding B cell subset. (**A**) Insulin-binding B cells can be found in all major B cell subpopulations, with increased frequency in the transitional and naïve (CD27−) subpopulations (populations 1, 3, 4, and 6). Tetanus-binding B cells are more enriched in the memory subpopulations (populations 2, 8) with few cells in the anergic subset (population 4). UMAP projections were generated using the unsupervised clustering algorithm, PhenoGraph, using the following markers: CD27, CD38, CD10, IgM, IgD, IgG, CXCR5, CD11c, and CD86. Antigen-binding populations were manually gated. (**B**) Representative heatmap of expression of various cell surface markers expressed on insulin, tetanus, and non-binding B cells. Colors represent raw mean metal intensities (MMI). Heatmap was generated using CellEngine.

One of the major advantages of mass cytometry is the large number of parameters that can be analyzed simultaneously, which allows for high dimensional reduction and unbiased monitoring of the immune system. There are many well-written reviews that discuss the various unsupervised clustering algorithms available to analyze mass cytometry data [23–25]. In Figure 4, we show an example of how the unsupervised clustering algorithm, PhenoGraph, can be used to identify various B cell subpopulations using UMAP projections and how the population makeup of insulin, tetanus, and non-binding B cells can be easily visualized and compared. Using this method, we can conclude that in this individual that insulin-binding B cells occur in almost all B cell subpopulations, but particularly in the transitional and naïve (CD27-) subpopulations (populations 1, 3, 4, and 6) and in the B_ND_ anergic population (population 4), while tetanus-binding B cells are enriched in the memory subpopulations (populations 2, 8) with few cells in the anergic subset (population 4) (Figure 4A). In addition, we can analyze the differences in surface marker expression of insulin, tetanus, and non-binding B cells by heat mapping, as discussed above (Figure 4B).

## 4. Discussion

Here we demonstrate that enrichment and characterization of antigen-binding B cells can be accomplished using magnetic nanoparticle adsorbents in conjunction with mass cytometry. Moreover, we show that simultaneous identification and analysis of more than one type of antigen-binding B cell is possible within the same sample. We believe this method will greatly inform our understanding of the phenotype and function of the pertinent antigen-reactive B cells during the course of the disease, such as in autoimmunity or cancer, as well as in infection, such as SARS-CoV2. The amount of information that can be gleaned from studying antigen-reactive B cells using mass cytometry data can lead to novel biomarker discoveries that can be used to predict and track the progression of disease and treatment outcomes.

While this method has its advantages over other techniques used to study antigen-reactive B cells, such as the ability to analyze many more parameters in one sample than one can achieve using flow cytometry and without the need for compensation or burden of possible autofluorescence, it does come with its own limitations. As discussed above, the low throughput of mass cytometry typically results in fewer cell events compared to flow cytometry, when starting from similar total cell numbers. One way to overcome this limitation is to start with more cells than you typically would for flow cytometry prior to enrichment. In addition, preliminary studies in our own lab have shown that the addition of “filler” cells labeled with a unique metal labeled anti-CD45 antibody mixed into the final enriched cell population can increase the number of enriched cells recovered for analysis.

Another limitation of this protocol is the limited availability of cleavable beads. Miltenyi Biotech currently offers anti-biotin, anti-FITC, anti-APC, and anti-PE cleavable beads. Due to the large size of APC and PE (105 kD and 250 kD, respectively), we prefer not to label our antigens with these fluorophores, since they potentially contain many antigenic epitopes that could result in increased enrichment of fluorophore-reactive B cells, as previously described [26]. Hence, the use of biotin and FITC, which are much smaller in size (0.244 kD and 0.389 kD, respectively), to label antigens of interest limits the number of antigens that can be concurrently enriched for. Ideally, one could just directly label an antigen with a metal isotope and avoid the use of secondary antibodies for detection, which may be possible depending on the antigen of interest and immune status of the individual. However, from our experience, detection of antigen-reactive B cells from both autoimmune subjects and recently vaccinated individuals requires prior enrichment. In addition, we find the addition of a secondary antibody enhances the signal generated, allowing for better discrimination of binding from non-binding B cells.

Other general limitations of studying B cells using mass cytometry include (1) the lack of ability to study the repertoire, which is currently best accomplished using single-cell sequencing, (2) the inability to determine the relevant affinity of the BCR for its antigen, which would require knowledge of the variable region sequences, generation of recombinant antibodies, and testing their affinity for their antigen using surface plasmon resonance, and (3) the destruction of the cells in the process, which prevents the ability to conduct downstream functional or in vivo studies. While no method to study antigen-reactive B cells has proven to be without its faults, once detection has been optimized using flow cytometry, as previously described [10], this method is quick, relatively simple, and results in reproducible high dimensional data that can be used to deeply probe the immune system on an antigen-specific level.

## Figures and Tables

**Table 1.** Materials.

Name:	Company:	Catalog Number:	Comments:
**LS Columns**	Miltenyi Biotech	130-042-401	
**Anti-Biotin Multi-sort Kit**	Miltenyi Biotech	130-091-256	
**Anti-FITC Multi-sort Kit**	Miltenyi Biotech	130-058-701	
**MACS manual separators**	Miltenyi Biotech	Variable	
**FcR Blocking Reagent human**	Miltenyi Biotech	130-059-901	
**Tetanus-toxoid**	Colorado Serum Company		Unadjuvanted with no carrier proteins present
**Human Insulin Biotinylated**	Eagle Biosciences	INS30-G100	
**PBS without calcium and magnesium**			No contact with autoclaved bottles or beakers or dish soap
**Ficoll-Paque PLUS**	VWR International	95021–205	
**Pierce^™^ FITC Antibody Labeling Kit**	ThermoFisher Scientific	53027	
**Pierce^™^ 16% Formaldehyde (*w*/*v*), Methanol-free**	ThermoFisher Scientific	28908	Dilute to 1.6% with CSM.
**Whole blood in heparinized collection tubes**			We typically use at least 30 mL of whole blood to enrich our cells of interest
**Cell Staining Media (CSM) (PBS + 0.5% BSA + 2 mM EDTA)**			No contact with autoclaved bottles or beakers or dish soap
**50 mL conical Tubes**			
**15 mL conical Tubes**			
**1.5 mL Eppendorf tubes**			
**MAXPAR-labeled antibodies of interest**			
**160Gd-anti-FITC antibody**	Fluidigm	3160011B	
**170Er-anti-biotin antibody**	Fluidigm	3170003B	
**Cisplatin live/dead**	Fluidigm	201195	Stock at 25 mM
**DNA Intercalator**	Fluidigm	201192B	500 uM stock

**Table 2.** Antibody panel.

Metal Isotopes:	Specificity:	Clone:	Vendor:
**113Cd**	CD45	HI30	Fluidigm
**115In**	CD3	HIT3a	Biolegend
**141Pr**	CD22	HIB22	Biolegend
**142Nd**	CD19	HIB19	Fluidigm
**143Nd**	CD5	UCHT2	Fluidigm
**144Nd**	CD69	FN50	Fluidigm
**145Nd**	CD95	DX2	Biolegend
**146Nd**	IgD	IA6–2	Fluidigm
**147Sm**	CD20	2H7	Fluidigm
**148Nd**	CD38	HIT2	Biolegend
**149Sm**	CD24	ML5	Biolegned
**150Nd**	CD86	IT2.2	Fluidigm
**151Eu**	HLA-DR	G46–6	Fluidigm
**152Sm**	CD36	5–271	Fluidigm
**153Eu**	CD8	SK1	Biolegend
**154Sm**	CD40	5C3	Biolegend
**155Gd**	CD27	L128	Fluidigm
**156Gd**	CXCR3	G025H7	Fluidigm
**158Gd**	CD10	HI10a	Fluidigm
**159Tb**	IgKappa	MHK-49	Biolegend
**160Gd**	Anti-FITC	FIT-22	Fluidigm
**161Dy**	CD80	2D10.4	Fluidigm
**162Dy**	CD11c	Bu15	Fluidigm
**163Dy**	CD72	3F3	Biolegend
**164Dy**	CD71	CY1G4	Biolegend
**165Ho**	CXCR5	J252D4	Biolegend
**166Er**	CD23	B3B4	Biolegend
**167Er**	IgLambda	1-155-2	Biolegend
**168Er**	CD138	DL-101	Fluidigm
**169Tm**	IgG	G18–145	BD Pharmingen
**170Er**	Anti-biotin	1D4-C5	Fluidigm
**171Yb**	PD-L1	29E.2A3	Biolegend
**172Yb**	IgM	MHM-88	Fluidigm
**173Yb**	CD21	Bu32	Biolegend
**174Yb**	CD4	SK3	Fluidigm
**175Lu**	CCR4	L291H4	Fluidigm
**176Yb**	PD-1	EH12.2H7	Biolegend
**191Ir**	Intercalator		Fluidigm
**193Ir**	Intercalator		Fluidigm
**195Pt**	Live-dead		Fluidigm
